# Finite element analysis of percutaneous uniplanar screw fixation in the treatment of thoracolumbar fractures

**DOI:** 10.1186/s40001-025-02785-z

**Published:** 2025-06-23

**Authors:** Shen Cao, Tao Xuan, Chengbao Zhang, Yubing Zhang

**Affiliations:** Department of Spine Surgery, Anhui No. 2 Provincial People’s Hospital, Hefei, China

**Keywords:** Finite element analysis, Uniplanar screw, Minimally invasive percutaneous fixation, Thoracolumbar fracture

## Abstract

**Objective:**

To compare the biomechanical characteristics of thoracolumbar fractures treated with uniplanar screws, monoaxial screws, and polyaxial screws using finite element analysis.

**Methods:**

CT data of the thoracolumbar spine T_12_–L_2_ from a healthy volunteer were collected, and using finite element software, models of both normal and fractured spines were created. Three different fixation models were constructed with monoaxial screws (Mps group), polyaxial screws (Pps group), and uniplanar screws (Ups group), respectively. The L_2_ vertebra was fixed and a compressive load of 150 N and a torque of 10 N•m were applied at the T_12_ end to simulate flexion, extension, lateral bending, and rotation movements of the spine. The range of motion (ROM) and internal fixation stress within the screws and rods were recorded for each movement direction.

**Results:**

A finite element model of the healthy human spine T_12_–L_2_ was established and validated for accuracy. All three fixation models demonstrated decreased ROM in all tested movements. The UPS group showed the lowest percentage of ROM in flexion, extension, and lateral bending movements, with a mid-range percentage of ROM in rotation, and relatively the best stability. The PPS group had the highest ROM percentages in all directions of movement, with the worst relative stability. The maximum von Mises stress for pedicle screws and rods in all fixation modes occurred in flexion, with the MPS group’s screws showing significantly higher stress peaks in flexion and both rotations than those of the PPS and UPS groups. The rods of the PPS group had significantly lower stress peaks in all motion states compared to those of the MPS and UPS groups.

**Conclusions:**

Uniplanar screws can effectively distribute stress, reduce the risk of screw and rod breakage, and ensure stability of spinal fixation.

## Introduction

With the rapid development of industries such as transportation and construction, the incidence of thoracolumbar fractures caused by car accidents and construction accidents is trending upwards annually, severely impacting patients'quality of life. Thoracolumbar fractures specifically refer to fractures at the T11–L2 segments, located at the junction of the thoracic and lumbar spine, where fractures are most likely to occur, accounting for about 60% of all spinal fractures [1]. For thoracolumbar fractures without nerve damage, early standard procedures used monoaxial pedicle screws (Mps) for fixation across the injured vertebrae. With the speedy advancement of spinal surgery techniques and the annual promotion of minimally invasive surgical concepts, minimally invasive percutaneous fixation for thoracolumbar fractures without nerve damage has gradually replaced conventional open posterior surgery as the clinical treatment of choice. Compared to traditional cut-open reset pedicle screw–rod fixation, minimally invasive percutaneous fixation can reduce surgical trauma, decrease intraoperative bleeding, and by avoiding extensive muscle detachment and burning during surgery, preserves the posterior ligamentous complex, which facilitates rapid postoperative recovery and early ambulation post-surgery. Additionally, it prevents postoperative paraspinal muscle denervation atrophy and fibrotic scarring, reducing the incidence of long-term postoperative iatrogenic back pain [2, 3].

However, percutaneous screw placement is limited by exposure and operational constraints, leading to the common use of polyaxial pedicle screws (Pps). These screws have a ball socket design allowing their tails to move in various directions around the rod, simplifying the procedure [4]. As they’ve been used clinically, polyaxial screws have been noted to have lower anti-bending strength and less load capacity compared to monoaxial screws [5], and their effectiveness in repositioning and maintaining vertebral height has also been reported as suboptimal [6]. In this context, uniplanar pedicle screws (Ups) were developed. Unlike traditional minimally invasive polyaxial screws, the tail of a uniplanar screw can only swing within the vertical plane of the connecting rod, with a swing range of − 25° to + 25°. This unique design not only facilitates rod placement, but also overcomes the issues of height loss and false distraction seen with conventional polyaxial screws. It combines the easy rod placement of polyaxial screws with the height retention benefits of monoaxial screws during distraction and compression to achieve better clinical outcomes.

Currently, there are many types of pedicle screws used clinically, and numerous combinations thereof. However, ethical restrictions limit the ability to directly conduct prospective studies on these screws and their combinations clinically. Unlike clinical studies, biomechanical experiments face no ethical restrictions and provide a reliable and accessible research approach. Finite element analysis, as a novel and effective biomechanical research technique, allows researchers to develop multiple internal fixation models for the same fracture model and conduct biomechanical analyses to evaluate stress concentration, stability, and repair effects under various fixations, providing valuable guidance for clinical treatment. This paper establishes a thoracolumbar fracture model using finite element methods, and constructs three groups of internal fixation finite element models using monoaxial screws, uniplanar screws, and polyaxial screws for stress analysis. It compares the stability and stress distribution of different fixation models and analyzes their respective biomechanical characteristics.

## Materials and methods

### Establishment of the normal model

A Siemens 64-slice spiral CT scanned from the upper edge of the T_12_ vertebra to the lower edge of the L_2_ vertebra on one volunteer, with each slice being 0.65 mm thick. The collected DICOM images were imported into Mimics17.0, where appropriate grayscale thresholds were set, and through processes such as threshold segmentation, dynamic region growth, Mask editing, and 3D calculation, the initial bony 3D geometric model was created. The STL files were processed using Geomagic Studio10.0 reverse engineering software to fix defects, optimize the structure, and produce a solid model with continuous surfaces. The facet joints and intervertebral discs were then reconstructed using the 3D CAD tools in SolidWorks2017 software. The built lumbar model was imported as a STEP file into Ansys19.0 for finite element preprocessing, setting up contact relationships between intervertebral discs, adjacent vertebral bodies, and facet cartilages, and dividing the mesh grid. Seven critical ligaments were modeled using nonlinear spring elements. Based on existing literature [7, 8], the Young’s modulus and Poisson ratio material coefficients for each were input into the model; their material properties are shown in Table 1.

**Table 1:** Material properties of the finite element models

Component	Young’s modulus (MPa)	Poisson ratio	Cross-sectional area (mm^2^)
Cortical bone	12000.0	0.3	–
Pedicle	3500.0	0.4	–
Cancellous bone	350.0	0.3	–
Endplate	500.0	0.4	–
Annulus fibrosus	4.0	0.4	–
Nucleus pulposus	1.0	0.5	–
Anterior longitudinal ligament	8.0	0.3	22.4
Posterior longitudinal ligament	12.0	0.3	7.0
Supraspinous ligament	8.0	0.4	22
Interspinous ligament	12.0	0.4	13
Intertransverse ligament	12.0	0.4	1.8
Ligamentum flavum	17.0	0.4	13.9
Joint capsule ligament	8.5	0.3	9.8
Titanium alloy	110000.0	0.3	–

### Establishment of fracture and fixation model

**Fig. 1:**
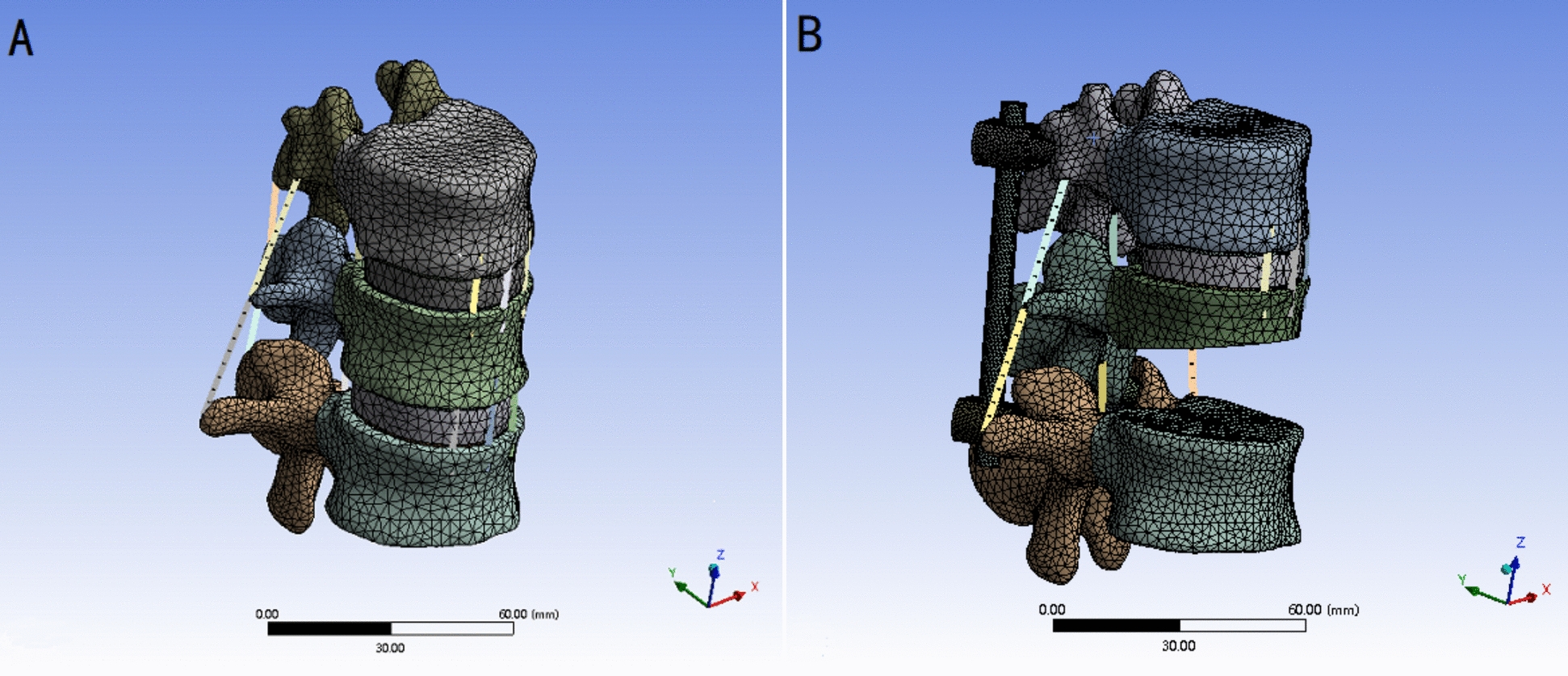
Finite element model. **A** T_12_–L_2_ normal spinal model. **B** Fracture and fixation model

To create a model close to clinical reduction fracture, we referenced the CT images from day 3 post-surgery of a patient with an L1 vertebral burst fracture. After reduction of the fractured vertebra, a wedge-shaped bone defect area appeared in the middle of the anterior column, comprising about 40% of the entire vertebra. Based on this radiographic change, we partially resected the L_1_ vertebra to simulate the actual injured vertebra. In order to simulate an extreme case of biomechanical instability similar to AO type A3.1 burst fracture, no anterior column implant was placed in the defect zone. This design allows for assessment of posterior screw systems under high-stress conditions. In SolidWorks, we imported the established model of the normal thoracolumbar spine, and drew a diagonal line from the midpoint of the anterior edge of the L_1_ vertebra to about two-thirds up the posterior edge. The portion of the vertebra below this line was resected in a wedge shape, while the posterior column structure was preserved, thus obtaining a model similar to the unstable A3.1 type thoracolumbar fracture in the AO classification (Fig. 1).

The pedicle screw system in this research was based on the spinal fixation system modeled from Fule Technology Development Co., Ltd. Three models of pedicle screws were created using software SolidWorks, with screws having a diameter of 6 mm, a length of 45 mm, and rods with a diameter of 6 mm. The pedicle screws were divided into three groups based on different specifications, forming three distinct fixation models. Monoaxial pedicle screws (Mps) were used for the Mps group, with the screw tail and rod being rigidly connected. This configuration utilized a fixation across the injured vertebrae, consisting of 4 screws and 2 rods. Polyaxial pedicle screws (Pps) were used for the Pps group, featuring a ball socket design at the screw tail for omnidirectional movement around the screw shaft, securing through the injured vertebra with 6 screws and 2 rods. Uniplanar pedicle screws (Ups) were used for the Ups group, featuring a unique rod design that restricts screw motion to a single plane perpendicular to the connecting rod, with a swing angle of − 25° to + 25°. Fixation was applied through the injured vertebra, involving 6 screws and 2 rods. The unique structures of the three different screws were modeled in SolidWorks and defined through constraints in Ansys software. The contact between the screw and bone was modeled as a bonded interface to simulate idealized screw fixation, ensuring a no-slip condition and allowing stress transmission across the interface.

### Finite element analysis

Import the three assembled final finite element models into Ansys finite element analysis software, and perform material property assignment, connection definition, mesh division, and finite element analysis. The material properties of vertebral bone, articular cartilage, endplate, nucleus pulposus, and titanium alloy internal fixation are set according to the finite element studies in previous literature, as shown in Table 1. After completing the assignment of material properties, definition of connections, and mesh division, the image coordinate system is set to match the motion direction of the lumbar spine, with the X-axis horizontal, the Y-axis anterior–posterior, and the Z-axis vertical. The X–Z axis represents the coronal plane of the lumbar spine, the X–Y axis represents the axial plane of the lumbar spine, and the Y–Z axis represents the sagittal plane of the lumbar spine. These loading values were chosen based on previous spinal finite element studies [7, 8], where 150 N axial force represents upper body weight, and 10 N·m torques represent physiological motion loads during flexion, extension, lateral bending, and rotation. Fix the lower part of the L_2_ vertebra and establish a reference plane, apply a pre-load of 150 N on T_12_ vertebra around the Z-axis to simulate the vertical compressive force on the lumbar spine from the skull direction. Then, apply a torque of 10 N•m on the positive X-axis to simulate left flexion, 10 N•m on the negative X-axis to simulate right flexion, 10 N•m on the positive Y-axis to simulate extension, 10 N•m on the negative Y-axis to simulate forward flexion, and apply 10 N•m torque clockwise and counterclockwise around the Z-axis to simulate left and right rotation of the lumbar spine.

### Evaluation indicators

Record the maximum angular displacement, also known as the range of motion (ROM), in each direction of motion for the three fixation models. Compare these with the ROM of the established normal thoracolumbar model to assess the percentage of mobility of the three fixation models relative to the complete model. Record the peak von Mises stress and stress distribution cloud diagrams for the pedicle screws and rods in the three fixation models during flexion–extension, lateral bending, and rotation. Since the model was based on only one subject, there is no need for statistical analysis of the results.

## Results

### Construction and validation

The T_12_–L_2_ normal spine model was established, fixing the lower part of the L_2_ vertebra and establishing a baseline plane. As previously described, we applied a compressive load of 150 N and a torque of 10 N•m to simulate lumbar flexion, extension, lateral bending, and rotational movements. The range of motion (ROM) were recorded for each direction of movement. For the T_12_–L_1_ segment, the ROM values are shown in Table 2. The results were comparable to those reported in the literature [7, 8]. The similarity of the study results confirms the validity of the model, allowing for further analysis.

**Table 2:** Finite element model and related literature ROM data comparison results

Motion	Literature	T_12_–L_1_	L_1_–L_2_
Flexion	4.0–6.3	4.13	4.36
Extension	3.4–6.7	3.66	4.21
Left lateral bending	3.7–5.3	5.07	4.85
Right lateral bending	3.7–5.3	5.18	5.02
Left rotation	1.6–3.8	3.11	3.35
Right rotation	1.6–3.8	2.88	2.96

### Range of motion (ROM)

**Fig. 2:**
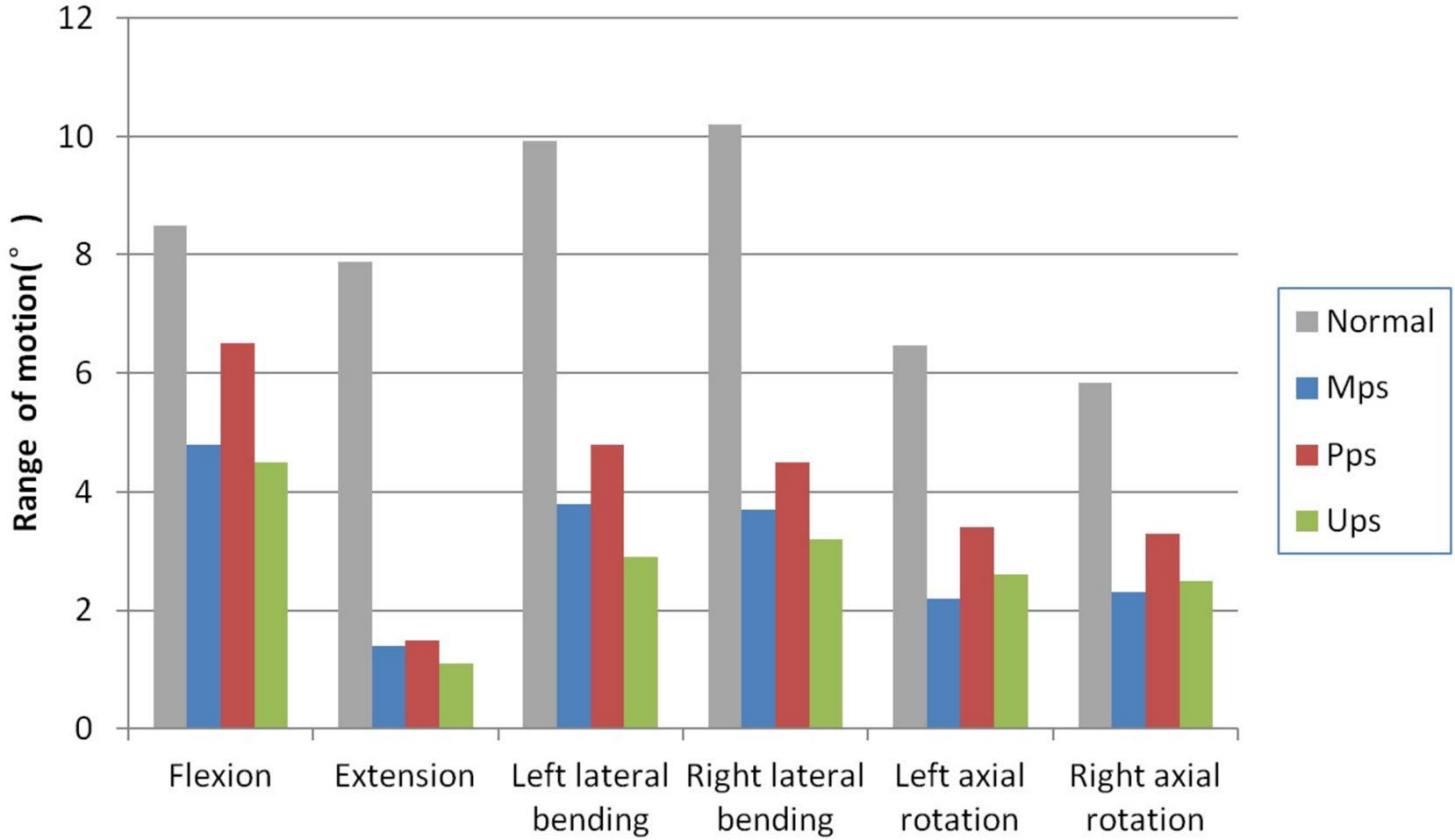
The results of range of motion (ROM)

Relative to the complete normal spinal model, all three fixation models exhibited a reduced ROM in the T_12_–L_2_ segments across all movement states, as shown in Fig. 2. The ROM percentages for each movement direction were compared among the three fixation models relative to the standard spine model. The UPS group had the lowest ROM percentages in flexion, extension, and lateral bending movements, with moderate ROM percentages in rotational directions (flexion: 53.0%; extension: 13.9%; left bend: 29.2%; right bend: 31.3%; left rotation: 40.2%; right rotation: 42.8%), offering relatively the best stability. The MPS group had the lowest ROM percentages in rotational movements, with moderate stability in ROM percentages in flexion, extension, and lateral bending (flexion: 56.5%; extension: 17.7%; left bend: 38.3%; right bend: 36.2%; left rotation: 34.0%; right rotation: 39.3%). The PPS group has the highest ROM percentages in all movement directions (flexion: 76.5%; extension: 19.0%; left bend: 48.3%; right bend: 44.1%; left rotation: 52.6%; right rotation: 56.5%), and has comparatively the worst stability (Fig. 2).

### Von Mises stresses

**Fig. 3:**
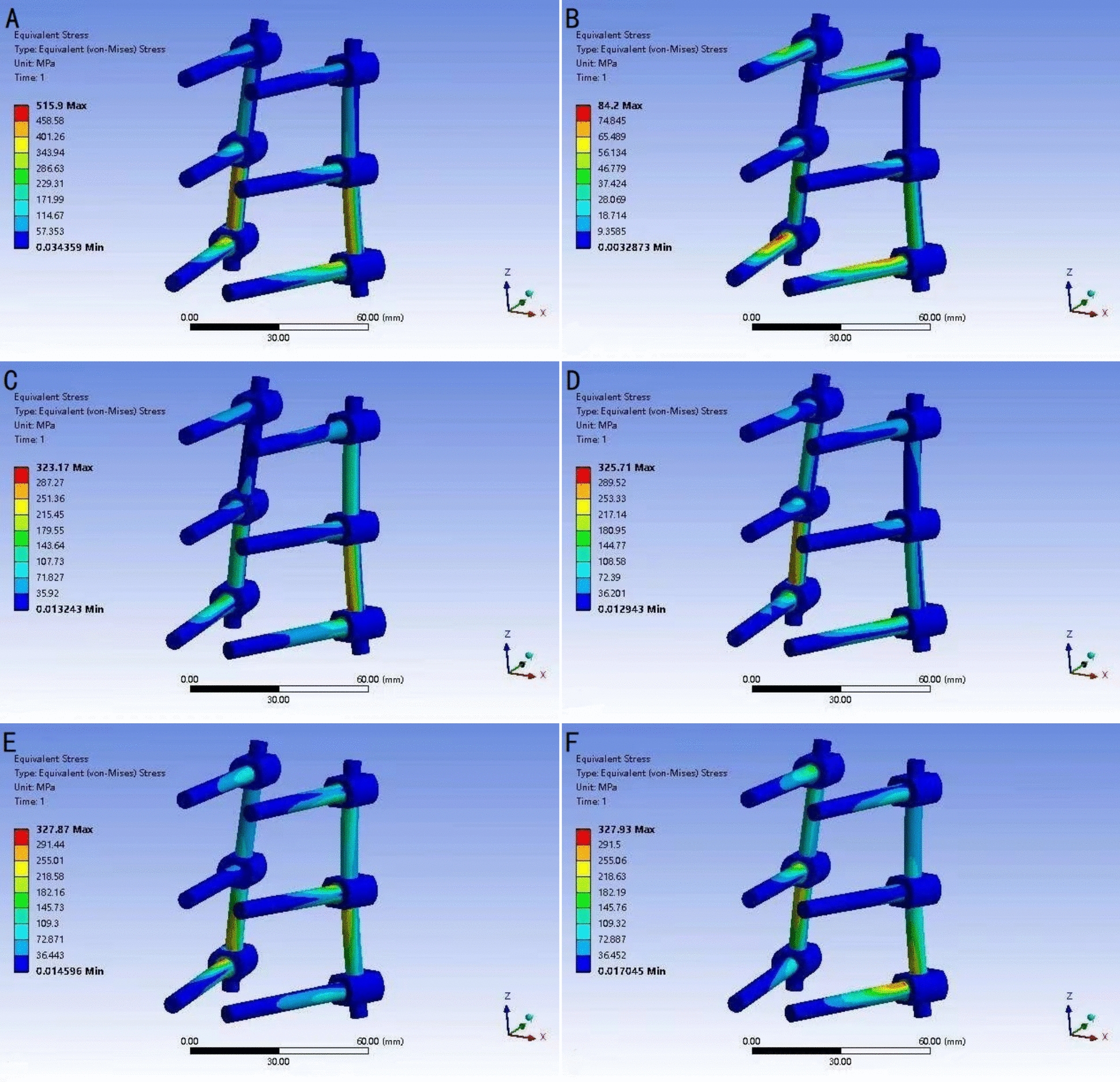
Stress distribution of the UPS group. **A** Flexion. **B** Extension. **C** Left lateral bending. **D** Right lateral bending. **E** Left rotation. **F** Right rotation

**Fig. 4:**
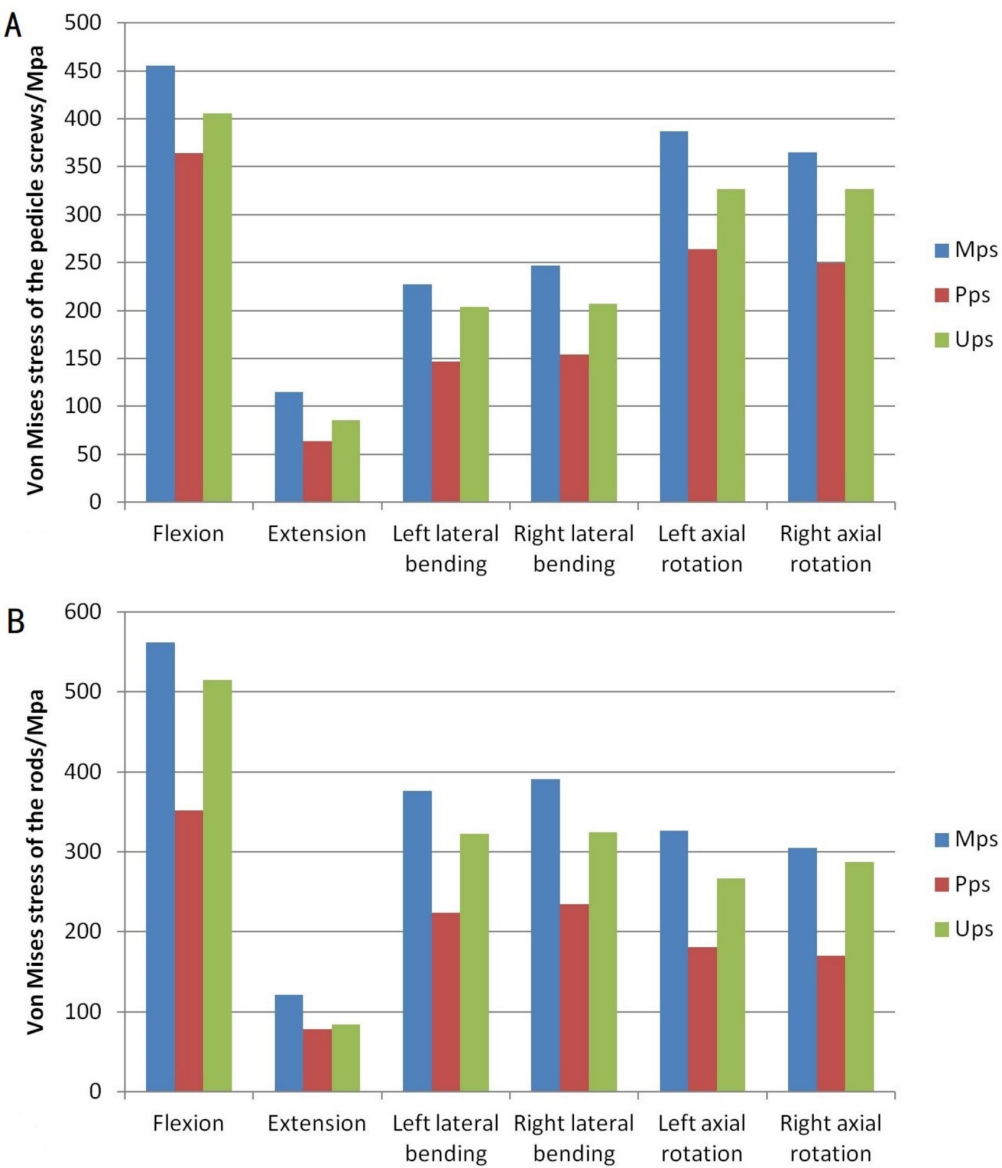
The peak von Mises stress. **A** Pedicle screws. **B** Rods

The von Mises stress distribution nephogram indicate that after lumbar spine fixation, the stress distribution under axial pressure changes, with screws and rods becoming the focal points of stress instead of the vertebrae. The peak stresses in the pedicle screws occur during flexion movements, and the greatest stress is found at the junctions between the pedicle screws and the rods (Fig. 3). In a lateral comparison of the three groups of pedicle screw models, the MPS group’s screw stress peaks are significantly higher than those of the PPS and UPS groups, especially in flexion and rotational movements. The peak stress values for screws are 455 MPa in the MPS group, 364 MPa in the PPS group, and 406 MPa in the UPS group (Fig. 4). For rod stress, in all fixation models under all movement conditions, the maximum von Mises stress always occurs during flexion and is located in the lower part of the rod. Specifically, the peak stress values for the PPS group in all movement states are significantly lower than those for the MPS and UPS groups. The peak stress values for the rods are 562 MPA in the MPS group, 352 MPa in the PPS group, and 515 MPa in the UPS group (Fig. 4).

## Discussion

Finite element analysis is an emerging and effective biomechanical research method, rapidly developed based on structural mechanics analysis. It offers high precision in mechanical analysis between measurement models and the interaction of loads and motion, thus it is favored over traditional animal and specimen experiments [9]. Finite element analysis has become increasingly refined in spinal biomechanics research, with models primarily established through geometric modeling, 3D coordinate instrument modeling, and image modeling. Typically, CT and MRI data of research samples are pre-collected and converted into compatible file formats before being inputted into finite element modeling software [10]. Finite element models can not only accurately reflect clinical realities and extensively simulate disease states, but also clarify the complex biomechanical characteristics of various bone structures. Analyzing spinal mobility and stress conditions across different movement states in various internal fixation models helps in selecting appropriate spinal fixation methods and predicting their outcomes [11].

In studies analyzing thoracolumbar fracture treatments using finite element analysis, establishing a correct finite element model is fundamental, yet currently, there is no uniform standard for constructing such models. Due to significant individual variations in fracture severity and soft tissue damage in clinical settings, some literature focuses on comparing changes in the elastic modulus of fractured vertebrae, damaged endplates, and adjacent intervertebral discs. However, differences in the location and extent of injuries mean changes in elastic modulus also vary greatly, making it challenging to accurately assign values to specific damaged tissues in analyses. Thus, current literature often resorts to physically removing parts of the vertebrae instead of reassigning values. Researchers have constructed models based on imaging changes observed immediately after thoracolumbar fractures or upon fixation. They found that after realignment of the fractured vertebrae, a wedge-shaped bone defect appeared in the middle of the anterior column, accounting for about 40% of the vertebra, leading to the removal of the central part of the vertebra in a wedge shape to establish the model. However, this method involves removing the vertebra at the pedicle plane. This paper, needing to study models with pedicle screws, opts to preserve the upper part of the vertebra for constructing the fracture model. Following the method described by Li [12], based on the AO classification's A3.1 type fracture, a diagonal line is drawn from the midpoint of the anterior edge to about two-thirds up the posterior edge, and the area below this line is removed in a wedge shape, representing about 40% of the entire vertebra, thus obtaining a model similar to the unstable A3.1 type thoracolumbar fracture. In this model, the lower half of L_1_ vertebral body and the L_1/2_ intervertebral disc are completely removed. The anterior column structure provides almost no support to the upper vertebrae, constituting severe instability. Through this extremely unstable spinal fracture model, we compare the stability and von Mises stress distribution of three types of pedicle screw internal fixation devices.

The thoracolumbar junction (T11–L2) is a transition zone between the rigid thoracic spine and the more flexible lumbar spine. This anatomical configuration results in a high incidence of burst fractures in this region. Additionally, clinical strategies for stabilizing thoracolumbar fractures remain controversial, particularly the choice between short-segment versus long-segment fixation. While long-segment constructs often provide more robust stabilization, they also increase invasiveness and reduce motion segments. This study focuses on short-segment constructs as they are more compatible with minimally invasive approaches.

Compared to the established normal spinal model, all three fixation models demonstrate reduced range of motion in all states of movement, indicating that all three internal fixations are effective and reliable. Among them, the Pps group shows the highest ROM percentage in all movement states, with relatively the poorest stability. This is related to the characteristics of the polyaxial screws, which are designed for ball-and-socket devices, allowing the screw tail to move in all directions around the screw–rod axis, facilitating percutaneous fixation during rod insertion. However, their capacity to resist bending and rotation is comparatively weak, resulting in stability that is even less than that of the Mps group with four-screw fixation. Furthermore, it has been clinically found that polyaxial screws also have the disadvantage of poor resetting and maintaining vertebral height. The Ups group has the smallest ROM in all movement states, thanks to the uniplanar screw combining the advantages of polyaxial screws and monoaxial screws. The tail end of the uniplanar screw is fixed in the sagittal plane, thus achieving the same longitudinal stability as monoaxial screws, with stronger longitudinal support and better prying reset effects than polyaxial screws. Simultaneously, the lateral swinging of the screw tail in the horizontal plane can achieve vertebral fixation in injured vertebrae. The fixation in injured vertebrae provides good three-point fixation, achieving strong fixation on three planes, avoiding the parallelogram effect and the hanging effect, thus enhancing spinal biomechanical stability [11, 13]. FengXu et al [14] have confirmed through biomechanical research that the anti-axial load strength of the uniplanar screw internal fixation system is close to that of the monoaxial screws fixation system, significantly higher than that of the polyaxial screws fixation system; compared to polyaxial screws, uniplanar screws can effectively reduce the risk of postoperative correction loss.

Under axial pressure, the central cartilaginous endplates and bilateral pedicle roots of the vertebrae in a complete spinal model bear significant stress. After screw implantation into the vertebrae, the original load distribution is altered, with the force on the lumbar spine primarily borne by the fixation system. The stress distribution map of pedicle screws shows that screws and rods bear most of the load, with noticeable stress concentration at the junction of the screw and rod, consistent with clinical reports of pedicle screw fractures typically occurring at the base of the screw [15, 16]. Under all motion conditions, the peak stress in pedicle screws and rods occurs in the flexed position, with the Mps group displaying the highest stress values in nearly all motion conditions, significantly higher than the other two groups. This is because both the Pps and Ups groups are fixed through injured vertebrae. The additional pedicle screws in fractured vertebrae can bear certain load stresses, thereby sharing the stress generated by internal fixation [17, 18]. In the MPS group, the screw head–neck junction consistently showed sharp stress peaks under flexion and rotation, while in the UPS group, the stress was more uniformly distributed along the rod–screw interface. This stress distribution effectively reduces the likelihood of screw fractures or loosening, as well as the occurrence of rod breakage. In the Pps group, the use of polyaxial screws results in more dispersed stress concentrations, thus having the lowest peak stress values in all motion states among the three groups [19], but this is achieved at the expense of model stability.

Uniplanar screws offer a promising balance between rigidity and rod accommodation, making them ideal for minimally invasive approaches to Type A3 thoracolumbar burst fractures. However, in fractures involving multidirectional instability or highly comminuted vertebral collapse, their fixed-axis limitation may pose challenges. These scenarios may require more flexible fixation strategies or supplementary anterior support.

Although finite element analysis is an effective method in the biomechanical study of the lumbar spine, it has its limitations, such as not considering the role of paraspinal muscles, some tissue material properties are not well understood, and the biomechanical characteristics of soft tissues are linearly simplified in the analysis. The use of linear elastic properties for bone and disc tissues in this model is a simplification aimed at enhancing computational feasibility and comparability across fixation types. Although such simplifications do not capture true viscoelastic or anisotropic tissue behavior, they are widely accepted in spinal FEA studies as sufficient for trend analysis of stress distribution and segmental mobility. Although these simplifications are made under reasonable assumptions, they inevitably affect the computational results of the model. However, the results obtained from finite element analysis are not precise values, but indicate a trend, and the overall mechanical trends shown by the simulation results do not undergo substantial changes due to the simplified material characteristics. While our finite element model is based on a single healthy subject, this design choice allowed for controlled comparison of screw biomechanics. However, we acknowledge this as a limitation and recognize the need for future studies involving subject-specific models that reflect diverse anatomical variations and fracture morphologies.

Given the observed biomechanical performance, placement of screws through injured vertebrae can reduce the stress load on screw–rod systems, thereby lowering the risk of screw and rod breakage, but the use of polyaxial screws may reduce spinal stability. Using uniplanar screws can disperse stress while ensuring that spinal stability remains unaffected. Uniplanar screws may serve as a promising option in percutaneous fixation, combining ease of rod placement with improved vertebral height retention. This study's conclusions offer mechanical reference for treating Type A3.1 spinal burst fractures. While the biomechanical results in this study may not be perfect, they offer solid biomechanical evidence for spine surgeons who looking to treat thoracolumbar fractures with minimally invasive percutaneous internal fixation.

## Data Availability

No datasets were generated or analysed during the current study.
